# The Distribution of *Listeria* in Pasture-Raised Broiler Farm Soils Is Potentially Related to University of Vermont Medium Enrichment Bias toward *Listeria innocua* over *Listeria monocytogenes*

**DOI:** 10.3389/fvets.2017.00227

**Published:** 2017-12-21

**Authors:** Aude Locatelli, Micah A. Lewis, Michael J. Rothrock

**Affiliations:** ^1^Egg Safety and Quality Research Unit, U.S. National Poultry Research Center, Agricultural Research Service, United States Department of Agriculture, Athens, GA, United States; ^2^Quality and Safety Assessment Research Unit, U.S. National Poultry Research Center, Agricultural Research Service, United States Department of Agriculture, Athens, GA, United States

**Keywords:** *Listeria monocytogenes*, *Listeria innocua*, pastured poultry, UVM enrichment medium, live production farms

## Abstract

The occurrence of *Listeria monocytogenes* has been widely investigated in the poultry production chain from the processing plant to the final product. However, limited data are available on *Listeria* species, including *Listeria monocytogenes*, in the poultry farm environment. Therefore, fecal and soil samples from 37 pastured poultry flocks from 10 all-natural farms over 3 years were assessed to determine the prevalence and diversity of *Listeria* within these alternative poultry farm environments using standard cultural and molecular methods. *Listeria* species were isolated in 15% of poultry farm samples and included *Listeria innocua* (65.7%), *L. monocytogenes* (17.4%), and *Listeria welshimeri* (15.1%). Additional multiplex PCR serotyping showed group 1/2a-3a to be the most dominant *L. monocytogenes* serovar group. Based on these results, monoculture growth experiments were conducted on four *Listeria* soil isolates (three *L. monocytogenes* isolates representing the three recovered serovar groups and one *L. innocua* isolate) to determine if culture medium [tripticase soy broth (TSB) and University of Vermont modified *Listeria* enrichment broth (UVM)], inoculum concentration (10^2^ or 10^5^ CFU/ml), or incubation temperature (20, 30, and 42°C) differentially affected these *Listeria* species. Overall, very few significant growth differences were observed between the behavior of the three *L. monocytogenes* isolates (representing the three recovered serovar groups) under the growth conditions tested. Alternatively, at 30°C in UVM with the lower inoculum concentration, the *L. innocua* isolate had a significantly shorter lag phase than the *L. monocytogenes* isolates. In coculture growth studies under these same incubation conditions, the lag phase of *L. innocua* and *L. monocytogenes* was similar, but the final concentration of *L. innocua* was significantly higher than *L. monocytogenes*. However, cocultures in UVM for high inoculum concentration did not show preferential growth of *L. innocua* over *L. monocytogenes*. These results indicate that the use of UVM as an enrichment medium may preferentially allow *L. innocua* to outcompete *L. monocytogenes* at low concentrations, biasing the *Listeria* prevalence from these farm samples toward *L. innocua* and potentially underreporting the presence of *L. monocytogenes* in these environments.

## Introduction

The genus *Listeria* is currently comprised of 17 species, including 11 *Listeria* species described since 2009 (1). However, the genus *Listeria sensu stricto* includes six species: *Listeria innocua, Listeria ivanovii, Listeria grayii, Listeria monocytogenes, Listeria seeligeri*, and *Listeria welshimeri*. These species are well documented and are known to be commonly found in different environments throughout the world (2–6). Among all the *Listeria* species, *L. monocytogenes* is recognized as one of the most important foodborne pathogens in many industrialized countries. This pathogen is responsible for listeriosis, a potentially fatal disease that may lead to abortion or serious cases of meningitis, encephalitis, and septicemia (7, 8). Although listeriosis infections are uncommon, mortality rates can reach 30% in at-risk population groups (9–11). In 2015 in the United States, *L. monocytogenes* was responsible for an estimated 116 cases of listeriosis, 111 hospitalizations, and 15 deaths (12).

*Listeria* species have been isolated from a wide variety of environments including nature (13) and urban areas (14), agricultural environments (15), food processing plants (16, 17), and retail food (18, 19). The occurrence of *Listeria* species is of special interest in the food production chain due to the significant threat that *L. monocytogenes* represents to public health (20, 21). Numerous studies have investigated the occurrence of *L. monocytogenes* in final products and in food-processing and retail environments, thought to be the main source of contamination for the final product (21, 22). However, limited information is available on *Listeria* prevalence in poultry farm production environments. In a farm-to-fork approach, it is necessary to assess the incidence of *L. monocytogenes* along the entire production chain and particularly at the primary production step (the farm environment), taking into account that it could be a potential source of this pathogen into food processing plants. Few studies have investigated and characterized *Listeria* species in the farm environment, with *L. innocua* being the predominant species found on grow-out farms, representing ≤78% of all isolated *Listeria* species (15, 23–26). Other species such as *L. ivanovii, L. monocytogenes, L. welshimeri*, and *L. seeligeri* have also been identified in environmental farm samples or chicken feces, but their detection remains infrequent (15, 23).

The initial isolation of *L. monocytogenes* may be difficult due to its low cell number within the larger indigenous microflora of environmental samples. Thus, the detection of *Listeria* species involves selective enrichment procedures. Numerous one-step and two-step enrichment broths have been described during the past 50 years (27), with the three most commonly used procedures being the (1) modified ISO 11290-1, (2) USDA-Food Safety Inspection Service (FSIS) Microbiology Laboratory Guide (MLG) method 8.10, and (3) U.S. Food and Drug Administration Bacteriological Analytical Method (FDA-BAM) method #10. Several studies have shown that the enrichment procedure can result in *L. monocytogenes* being overgrown by other non-pathogenic *Listeria* species in samples where multiple species are present (28–32). This has especially been demonstrated with *L. innocua*, whose presence may mask *L. monocytogenes* and lead to false negative results (33, 34). In addition, several studies have shown that among *Listeria* strains of food origins, *L. innocua* grows faster than *L. monocytogenes* in enrichment media cocultures or food matrices (28–32). These observations raised the question whether the higher prevalence of *L. innocua* observed in samples from the farm environment is due to a differential growth of *Listeria* species during the enrichment process or reflect their true distribution in the environment.

While commercial, conventional production represents the majority of the U.S. poultry market, alternative production systems (e.g., organic, all-natural) are becoming more prevalent and there is very limited information related to the prevalence of *Listeria* spp. within this type of farm environment (35). Therefore, the goal of this work was twofold: (1) determine the prevalence and distribution of *Listeria* spp. within poultry-related environmental samples (feces and soil) during live production on pastured poultry farms and (2) evaluate whether the distribution of recovered *Listeria* spp. could be explained by a differential growth in the enrichment broth used in this study, or accurately reflected the native species distribution in the environment.

## Materials and Methods

### Sample Collection

Ten farms within the southeastern United States were sampled over a period of 3 years (from 2014 to 2016), representing 37 pasture-raised broiler flocks. Farm descriptions are available in Table 1. Soil and feces samples were collected from the pasture where the flock was currently residing at the time of sampling. Samplings occurred three times during grow-out: (i) within a few days of being placed in the pasture, (ii) halfway through their time on pasture, and (iii) on the day the flock was processed. At each sampling time, the pasture area was divided into five separate sections, and five subsamples in each section were pooled into a single sample for each section (a total of five soil samples and five feces samples were collected on each sampling day). Soil samples were collected from the surface (0–7 cm) with sterile scoops, and feces samples were collected from fresh droppings on the soil surface. Gloves and scoops were changed between sample types and between sampling areas. Samples were transported back to the lab on ice and processed within 2 h of collection. A total of 1,110 samples (555 feces samples and 555 soil samples) were collected over the 3-year study period.

**Table 1:** Characteristics of the 10 all-natural pastured poultry farms sampled over the 3-year period.

	Farm A	Farm B		Farm C	Farm D	Farm E	
Breed	Freedom ranger	Freedom ranger	Cornish cross	Freedom ranger	Freedom ranger	Freedom ranger	Cornish cross
Flock size	>500	50–75	50–75	50–75	50–75	50–75	50–75
No. of flocks	10	3	2	1	1	1	4
Length of grow-out (weeks)	10–11	13	13	12.5	11	11	9
Multiuse farm?	Yes	Yes	Yes	No	No	Yes	Yes
Animal types	Layers, swine, beef cattle, and sheep	Layers, swine, horses, and goats	Layers, swine, horses, and goats	n/a	n/a	Layers, swine, beef cattle, and sheep	Layers, swine, beef cattle, and sheep

	**Farm I**		**Farm J**		**Farm K**	**Farm L**	**Farm M**

Breed	Freedom ranger	Cornish cross	Freedom ranger	Cornish cross	Freedom ranger	Freedom ranger	Cornish cross
Flock size	100–500	100–500	50–75	50–75	100–500	>500	50–75
No. of flocks	5	3	1	1	2	2	1
Length of grow-out (weeks)	11–12	9	11	9	11	11–12	11
Multiuse farm?	Yes	Yes	Yes	Yes	Yes	Yes	Yes
Animal types	Layers, swine, and goats	Layers, swine, and goats	Layers	Layers	Layers, beef cattle, and goats	Layers, swine, beef cattle, and sheep	Layers and swine

### Culture-Based Detection and Isolation of *Listeria* Species from Soil and Feces Samples

Enrichment and isolation of *Listeria* from these environmental samples were performed using a modified version of the USDA-FSIS MLG 8.10 method (36). Three grams of fresh soil or feces were added to 9 ml of buffered peptone water (Acumedia, Lansing, MI, USA) in a filtered stomacher bag and were vigorously shaken for 30 s. As a pre-enrichment step, the stomached homogenates remained in the filtered stomacher bag and were incubated overnight at 35°C. This pre-enrichment step was followed by two enrichments in University of Vermont Modified *Listeria* Enrichment Broth (UVM; Remel, Lenexa, KS, USA) and Fraser Broth (Oxoid CM0895, Basingstoke, UK), both requiring overnight incubation for 24 h at 30°C. One loopful of the Fraser’s enrichment culture was streaked on *Listeria* selective agar (LSA, Oxoid CM0856, Basingstoke, UK) for the isolation of *Listeria* colonies. These plates were incubated overnight at 30°C, and on each plate three *Listeria*-like colonies per positive samples were picked and kept for further identification tests. Stock cultures were prepared by growing *Listeria* strains in tripticase soy broth (TSB; Acumedia, Lansing, MI, USA) at 37°C. After washing in sterile water, the cell pellet was suspended in a brain heart infusion (BHI) broth (Acumedia, Lansing, MI, USA) with 25% of glycerol, aliquoted (300 µl in microtubes) and frozen at −80°C until further utilization.

### Characterization of *Listeria* Species and *L. monocytogenes* Serovar Groups by Multiplex PCR

The species of presumptive *Listeria* colonies recovered on LSA were determined by multiplex PCR (37). In short, speciation occurred using two multiplex PCR reactions, based on the size of PCR amplicons. Pool 1 contained the primers for the identification of *L. ivanovii, L. grayi*, and *L. innocua*, and pool two contained primers for the identification of *L. welshimeri, L. monocytogenes*, and *L. seeligeri*. A 25 µl PCR reaction was composed of 1× EconoTaq PLUS 2× Master Mix (Lucigen Corporation, Middleton, WI, USA), 1 µM of each *livN, Igr*, and *lin2* reverse and forward primers (for Pool 1) or 1 µM of each *lwe, Lmo*, and *lse* reverse and forward primers (for Pool 2) and quantity sufficient (qs) of water. For negative controls, sterile water was added instead of template DNA. The cycling program consisted of 1 cycle at 95°C for 9 min; 30 cycles at 94°C for 30 s, at 60°C for 30 s, and at 72°C for 1 min; and 1 cycle at 72°C for 7 min. The serovar group of isolates classified as *L. monocytogenes* was determined by multiplex PCR using five sets of primers (38). Briefly, one colony of *L. monocytogenes* isolates was thoroughly mixed in a 25 µl PCR reaction containing: 1× EconoTaq PLUS 2× Master Mix (Lucigen Corporation, Middleton, WI, USA), 1 µM of each *Lmo0737, ORF2819*, and *ORF2110* reverse and forward primers, 1.5 µM of *Lmo1118* reverse and forward primers, 0.2 µM of *prs* reverse and forward primers and qs water. For negative controls, sterile water was added instead of template DNA. PCR was performed with an initial denaturation step at 94°C for 3 min; 35 cycles of 94°C for 0.40 min, 53°C for 1.15 min and 72°C for 1.15 min; and 1 final cycle for 72°C for 7 min. PCR reactions were performed in an Eppendorf Mastercycler EP Gradient S (Eppendorf). After the completion of all cycles, 18 µl of PCR product was mixed with 3 µl of BlueJuice™ loading buffer (Invitrogen, Carlsbad, CA, USA) and separated on a 2% E-gel^®^ with SYBR-safe™ (Invitrogen) along with 12 µl of E-Gel™ 1 kb Plus DNA Ladder (Invitrogen).

### Bacterial Growth Experiments

#### Bacterial Strain Selection and Inoculum Preparation

Based on the two main *Listeria* species found from farm distribution data, three *L. monocytogenes* strains representing the different recovered serovar groups (1/2a-3a, 1/2b-3b-7, and 4b-4d-4c) and one *L. innocua* were selected for the growth experiments. Pre-cultures were prepared by inoculating 60 ml of TSB with 100 µl of the thawed stock culture and incubated for 24 h at 30°C while shaking (150 rpm). After 24 h, cell density was estimated spectrophotometrically by measuring the optical density (OD) at 600 nm (OD_600nm_) with the Thermo Scientific Spectronic 200™ (Fisher Scientific). Pre-cultures were initially diluted in TSB or UVM to a concentration of 10^6^ CFU/ml, and then serially diluted in TSB or UVM to obtain final inoculum concentrations of 10^5^ and 10^2^ CFU/ml.

#### Monoculture Growth Experiments of *Listeria* Strains

A volume of 0.4 ml of each culture (10^5^ and 10^2^ CFU/ml) for the four *Listeria* strains was aliquoted into wells of a microplate (Honeycomb 2 cuvette plate; Labsystems, Inc., Franklin, MA, USA), with five repeats of each culture condition (strain × medium × concentration) per plate. Negative controls consisted on 0.4 ml of uninoculated TSB and UVM (five repeats) incubated along the cultures. For each culture, two independent plate repeats containing all treatment combinations were performed. The inoculated microplate was placed in a Bioscreen C microbiology reader (Thermo Electron Corp., West Palm Beach, FL, USA), which was operated by a computer with Growth Curves Software, v 2.28 (Transgalactic Ltd., Helsinki, Finland). The microbiology reader recorded the OD values of cultures at 20-min intervals after a plate shaking of 10 s at a medium speed (30 shakes/min). Three incubation temperatures were chosen, and the corresponding incubation times were adjusted to make sure that all growth curves reach the stationary phase by the end of the experiment. Plates were incubated at (i) 20°C, estimated soil temperature calculated upon the average of the atmospheric temperatures encountered during the sampling period (from March to August), for 48 h, (ii) 30°C, recommended temperature used for the enrichment procedure of *Listeria* spp. in UVM medium, for 24 h, and (iii) 42°C, expected temperature inside the chicken intestine, for 24 h.

#### Coculture Growth Experiments of *L. monocytogenes* Serovar Group 1/2a-3a and *L. innocua*

Since no significant growth differences were observed among the three *L. monocytogenes* isolates in the monoculture growth study, subsequent coculture growth studies with the *L. innocua* isolate were only performed with the *L. monocytogenes* 1/2a-3a isolate (the most prevalent serovar group found within the farm data). Three different coculture mixtures were used to observe coculture growth effects: (1) *L. monocytogenes* 1/2a-3a to *L. innocua* ratio of 10^2^:10^2^ CFU/ml in UVM, (2) *L. monocytogenes* 1/2a-3a to *L. innocua* ratio of 10^5^:10^5^ CFU/ml in UVM, and (3) *L. monocytogenes* 1/2a-3a to *L. innocua* ratio of 10^2^:10^2^ CFU/ml in TSB. As positive controls, monocultures of *L. monocytogenes* 1/2a-3a and *L. innocua* were tested under the same conditions (medium × concentration) as the coculture mixtures, while negative controls consisted of uninoculated TSB or UVM, respectively. Each coculture mixture was inoculated individually into a microplate along with positive and negative controls with a final inoculum volume of 0.4 ml for each condition. The OD was recorded at 30-min intervals after a brief plate shaking of 10 s at a medium speed (30 shakes/min) during the incubation at 30°C for 24 h. To quantify the growth of *L. monocytogenes* 1/2a-3a and *L. innocua* in coculture, 100 µl aliquots were sampled every hour from the microwell plate and serially 10-fold diluted. Appropriate dilutions were plated on Rapid’*L.mono* medium (Bio-Rad, Hercules, CA, USA). Blue and white colonies were enumerated as *L. monocytogenes* 1/2a-3a and *L. innocua*, respectively.

### Modeling the Microbial Growth Kinetics and Statistical Analysis

Growth curves were plotted based on OD values over time. Each bacterial growth curve was fitted to a modified Gompertz model using Matlab 2007b. The model equation is as follows:

$$y={Ae}^{\left\{-e^{\left[\frac{\mu_{\text{max}}\cdot e}{A}(\lambda -t)+1\right]}\right\}},$$

where *y* is the OD value measured, *t* is the time (h), μ_max_ is the maximum specific growth rate (h^−1^), *A* is the maximum OD value attained, and λ is the lag time (h). Within the m-file written in Matlab, the lsqcurvefit function (a nonlinear least-squares solver for data fitting) was utilized to fit the growth curves by first using the following Gompertz equation:

$$y=Ae^{-e(B-Cx)}.$$

Then, the *A, B*, and *C* terms were used to determine the growth parameters of interest in the model equation as follows:

$${\text{μ}}_{m}=\frac{A\;\cdot \;C}{e},$$

$$\text{λ}=\frac{B-1}{C}.$$

The raw data for each growth curve were graphed along with the resulting fit, and the *R*^2^ value (coefficient of determination) for each resulting fit was calculated. A four-way analysis of variance (ANOVA) was performed separately on each growth parameter (λ, μ_max_, and *A*), followed by Tukey’s *post hoc* test in R software v3.2.1. Factors included in the model were the *Listeria* strains, the culture medium, the inoculum concentration, and the incubation temperature. For the coculture experiments, μ_max_ and stationary phase cell densities (equivalent to OD_max_) were log10-transformed before ANOVA, and a Tukey’s *post hoc* test was used to group treatments. For all analyses, differences among groups were considered significant if *p* ≤ 0.05.

## Results and Discussion

### Prevalence and Distribution of *Listeria* Species in Soil and Feces Collected from Pastured Poultry Farms

**Figure 1:**
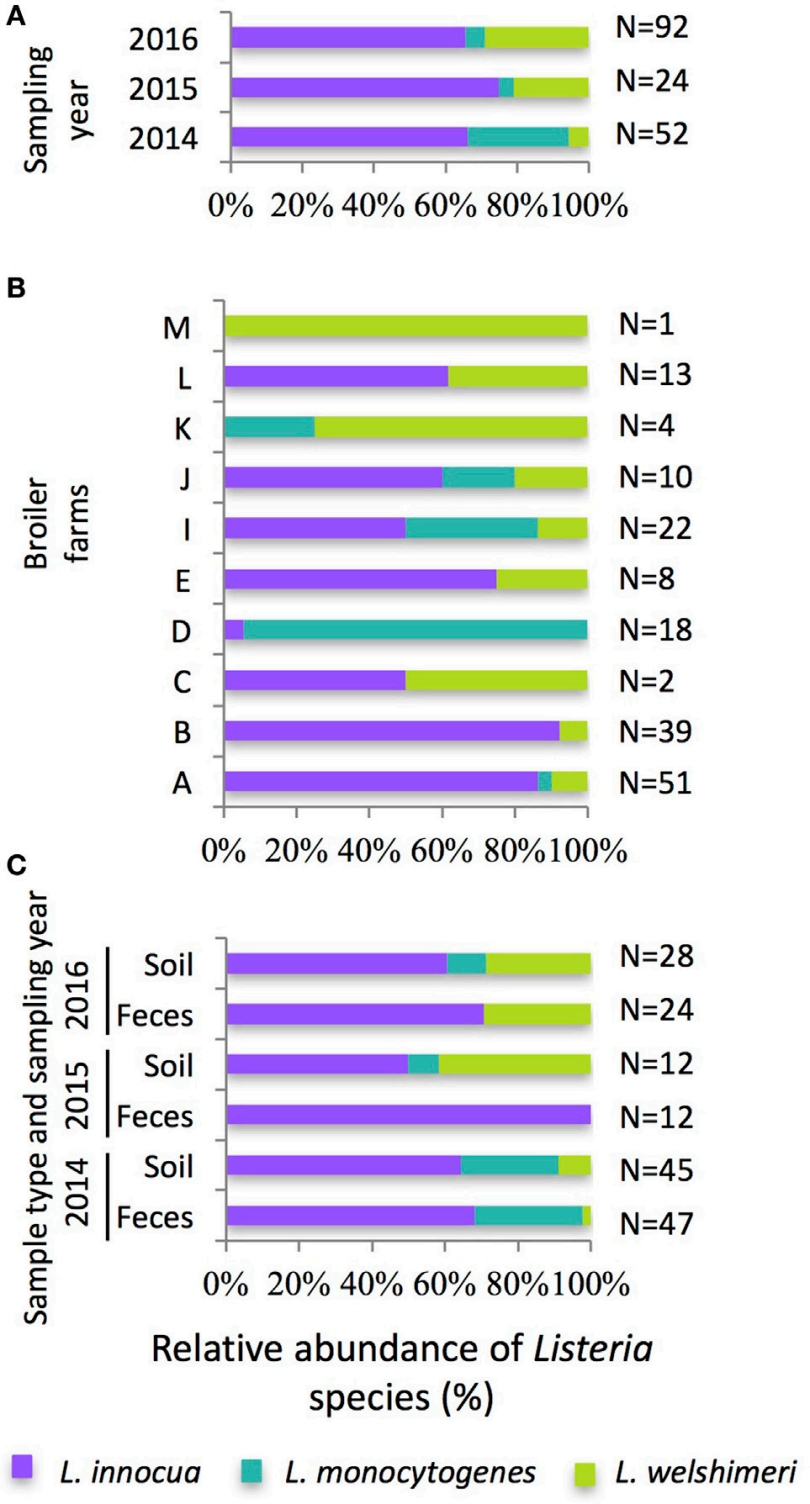
Relative abundance (% total *Listeria* species isolated) and distribution of the isolated *Listeria innocua, Listeria monocytogenes*, and *Listeria welshimeri* **(A)** according to the sampling year, **(B)** the broiler farm, and **(C)** the sample type over the 3-year sampling period. The number of *Listeria* species isolated per year/farm/sample type is indicated to the right of the bar.

A total of 1,110 samples (555 feces samples and 555 soil samples) were collected from 37 flocks on 10 pastured poultry farms over a 3-year period, and the distribution of *Listeria* species varied according to the sampling year (Figure 1A), broiler farm (Figure 1B), and sample type (Figure 1C). Overall, *Listeria* species were detected on all the farms and isolated in 15% of samples (83 from feces and 85 from soils), which is in the range of *Listeria* species prevalences reported in poultry-related environmental samples (from 1.4 to 53%) such as broiler litter, farm feed, farm drinking water, soil, and grass (25, 26, 39, 40) as well as in poultry feces (4.7–17%) (23, 25). In our study, three species were isolated including *L. innocua* (65.7%), *L. monocytogenes* (17.4%), and *L. welshimeri* (15.1%), and each of these species were recovered from at least half of the broiler farms (80, 50, and 90%, respectively; Figure 1B). Although different *Listeria* species distributions were observed between farms, at least two *Listeria* species were recovered from all but one farm (Farm M), and all three species were recovered from soil samples in all 3 years of the study (Figure 1C). *Listeria innocua* has been previously shown to be the predominant species isolated from the broiler farm environment (23–26, 40), while the detection of other non-pathogenic *Listeria* species, such as *L. welshimeri*, remains infrequent (23, 40), mostly because studies only focus on *L. monocytogenes* (41–43).

*Listeria monocytogenes* was isolated from 5.8, 0.3, and 1.0% of all samples collected in 2014, 2015, and 2016, respectively, with 87% (26/30) recovered during 2014 (Figure 1A) and 57% (17/30) of all *L. monocytogenes* isolates coming from the only flock sampled on Farm D in 2014 (Figure 1B). Overall, three *L. monocytogenes* serovar groups were identified: 1/2a-3a (70%), 1/2b-3b-7 (20%), and 4b-4d-4e (10%). Interestingly, over the 3-year sampling period, only one *L. monocytogenes*-positive flock (Farm I, 2014) harbored more than one serotype, demonstrating the potential clonal nature of *L. monocytogenes* within a flock or on a farm (44). The overall prevalence of *L. monocytogenes* on these 10 farms was low compared with other grow-out farm environments where 0–46.2% of the environmental and feces samples were *L. monocytogenes* positive (45), but the distribution of the serotypes was consistent with other studies that have characterized *L. monocytogenes* serotypes in broiler flocks (26, 41, 43). The prevalence of *L. monocytogenes* contamination may be dependent on the type of production system. A significant difference between caged- and floor-reared hens was observed with a greater detection of *L. monocytogenes* in dust samples from floor-reared hens in *L. monocytogenes*-positive flocks (41). In alternative systems, broilers are raised in less controlled environments than conventional systems and are more likely to be in contact with *L. monocytogenes* known to be widely spread in soil and vegetation (35).

Poultry farms frequently have other animals (beef cattle, sheep, goats, or swine) and pets present on the production site (35). These animals can be reservoirs for and play a role in the proliferation and deposition of *L. monocytogenes* into the environment. In our study, all but two farms had other animals raised in close proximity to the broiler flocks during the sampling period, but we did not investigate the possible genotype matching between animal species. Generally, the presence of other animals on the farm increase the risk factor associated with pathogenic bacteria contamination of poultry flocks (42, 46). This has been shown with *Campylobacter* spp. where adjacent broiler flocks and cattle appear to be the most frequently identified animals with broiler-flock matching *Campylobacter* spp. isolates (47). Another study has reported an increased risk of *L. monocytogenes* contamination in laying hen flocks when pets were present on the production site (42).

### Monoculture Growth Experiments of *L. innocua* and *L. monocytogenes* Isolated from Pastured Poultry Farm Soils

#### Growth Curve Modeling and Determination of Bacterial Growth Parameters

Our field results showed a higher prevalence of *L. innocua* compared with *L. monocytogenes* on pastured poultry grow-out farms, which has been supported by other studies reporting the incidence and characterization of *Listeria* species in the commercial poultry farm environment (15, 23, 24, 26). In terms of food safety interests, there is a question as to whether there is any physiological basis for the dominance of *L. innocua* over *L. monocytogenes* within the poultry farm environment, and whether this dominance related to preferential growth. To determine if this environmental dominance of *L. innocua* over *L. monocytogenes* may be linked to growth conditions (e.g., initial concentration, growth temperature, and growth medium), monoculture and coculture growth studies were performed. Three *L. monocytogenes* isolates (one strain of each serovar groups: 1/2a-3a, 1/2b-3b-7, and 4b-4d-4e) and one *L. innocua* isolate were selected to compare their growth capacity in liquid media. Bacterial growth was monitored by recording the OD of a culture in growth media (TSB and UVM) inoculated at different initial concentrations (10^2^ and 10^5^ CFU/ml) and incubated at 20°C (average environmental temperature), 30°C (UVM enrichment temperature according to USDA-FSIS MLG 8.10), or 42°C (broiler body temperature). Curve modeling was performed with the Gompertz function that fits the data with *R*^2^ values ranging from 0.674 to 0.998, indicating a good fit. From the modified Gompertz equation, three relevant parameters [lag time (λ), maximum specific growth rate (μ_max_), and maximum OD (OD_max_)] were determined for each curve and subsequently used to statistically compare the bacterial growth of the *Listeria* strains under the different cultural conditions. Using a four-way ANOVA (Tables S1–S3 in Supplementary Material for λ, μ_max_, and OD_max_, respectively), we investigated whether the culture medium, the inoculum concentration, and the incubation temperature could explain the global variation observed between the growth curves. In the same model, we more specifically examine the growth differences between the four *Listeria* isolates for a single culture condition. The parameters λ, μ_max_, and OD_max_ representing bacterial growth characteristics were used in the model.

#### Effect of Culture Medium, Inoculum Concentration, and Incubation Temperature on Lag Time (λ)

**Figure 2:**
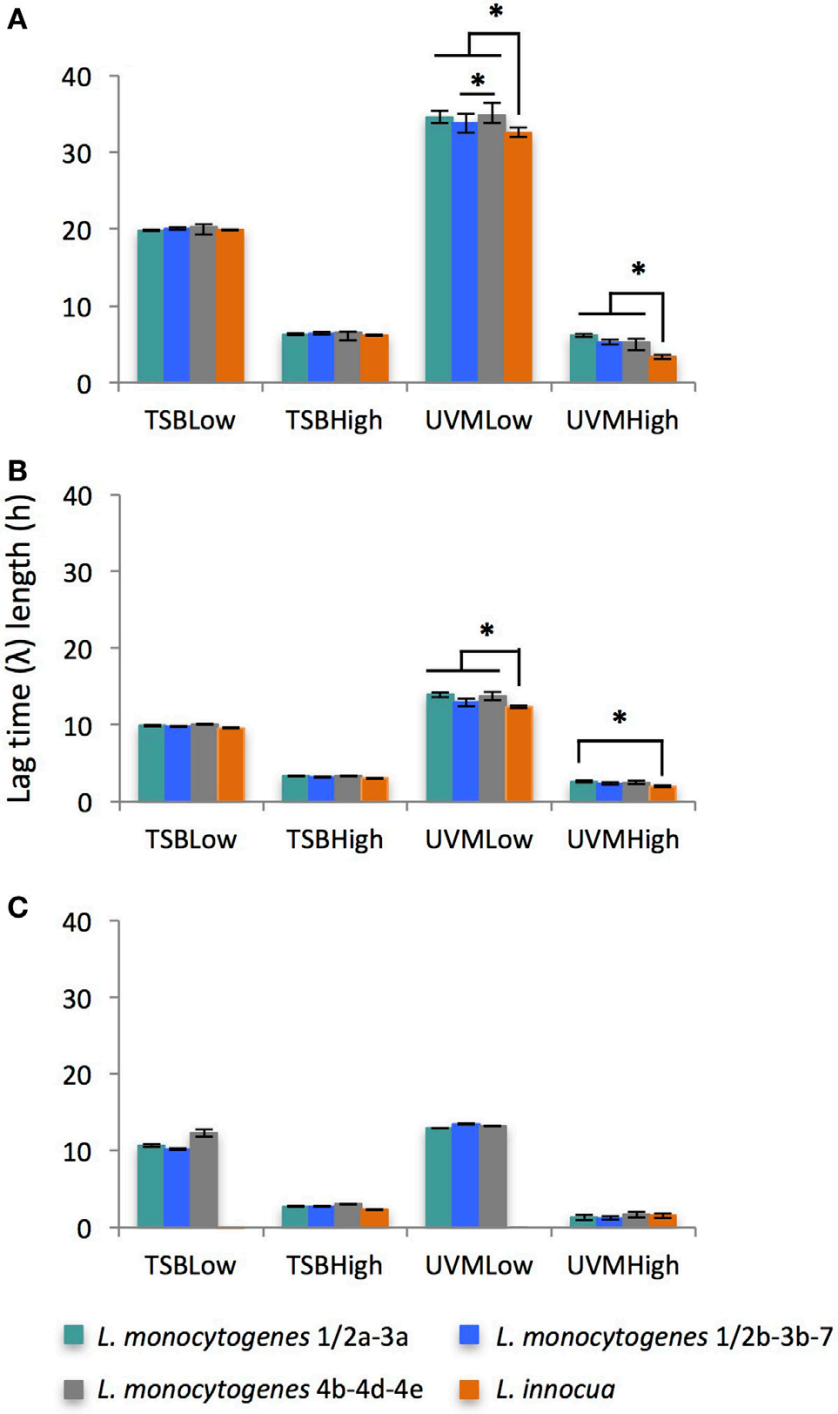
Average of lag time (λ) length of *Listeria monocytogenes* and *Listeria innocua* strains inoculated at low (10^2^ CFU/ml) and high (10^5^ CFU/ml) concentrations in TSB and UVM incubated at **(A)** 20°C, **(B)** 30°C, and **(C)** 42°C. *Significant differences between strains (*p* < 0.05). No growth observed for *L. innocua* in TSBLow and UVMLow at 42°C.

Unsurprisingly, λ was significantly shorter for the higher inoculum concentrations for all strains, enrichment media, and incubation temperatures (*F* = 801, *p* < 0.0001; Figure 2). This is in agreement with other studies that have evidenced the importance of inoculum concentration on the ability of a microbial population to initiate growth (48, 49). While Baranyi et al. showed that as the cell numbers in the inoculum decrease, λ increases (50, 51), other studies have reported an effect of the inoculum size only under stressful conditions (49, 52). Increasing incubation temperature significantly decreased λ (*F* = 174, *p* < 0.0001) in both TSB and UVM enrichment media for all *Listeria* strains, as has been showed in other growth media for both *L. monocytogenes* and *L. innocua* (28, 49, 52, 53). The temperature-dependent effect was significantly greater in the low initial concentration treatments compared with the higher initial inoculum treatments (*F* = 77, *p* < 0.0001). While lag time was significantly shorter in TSB compared with UVM, this was the weakest association of the major growth variables tested (*F* = 60, *p* < 0.05).

#### Effect of Culture Medium, Inoculum Concentration, and Incubation Temperature on Maximum Specific Growth Rate (μ_max_)

**Figure 3:**
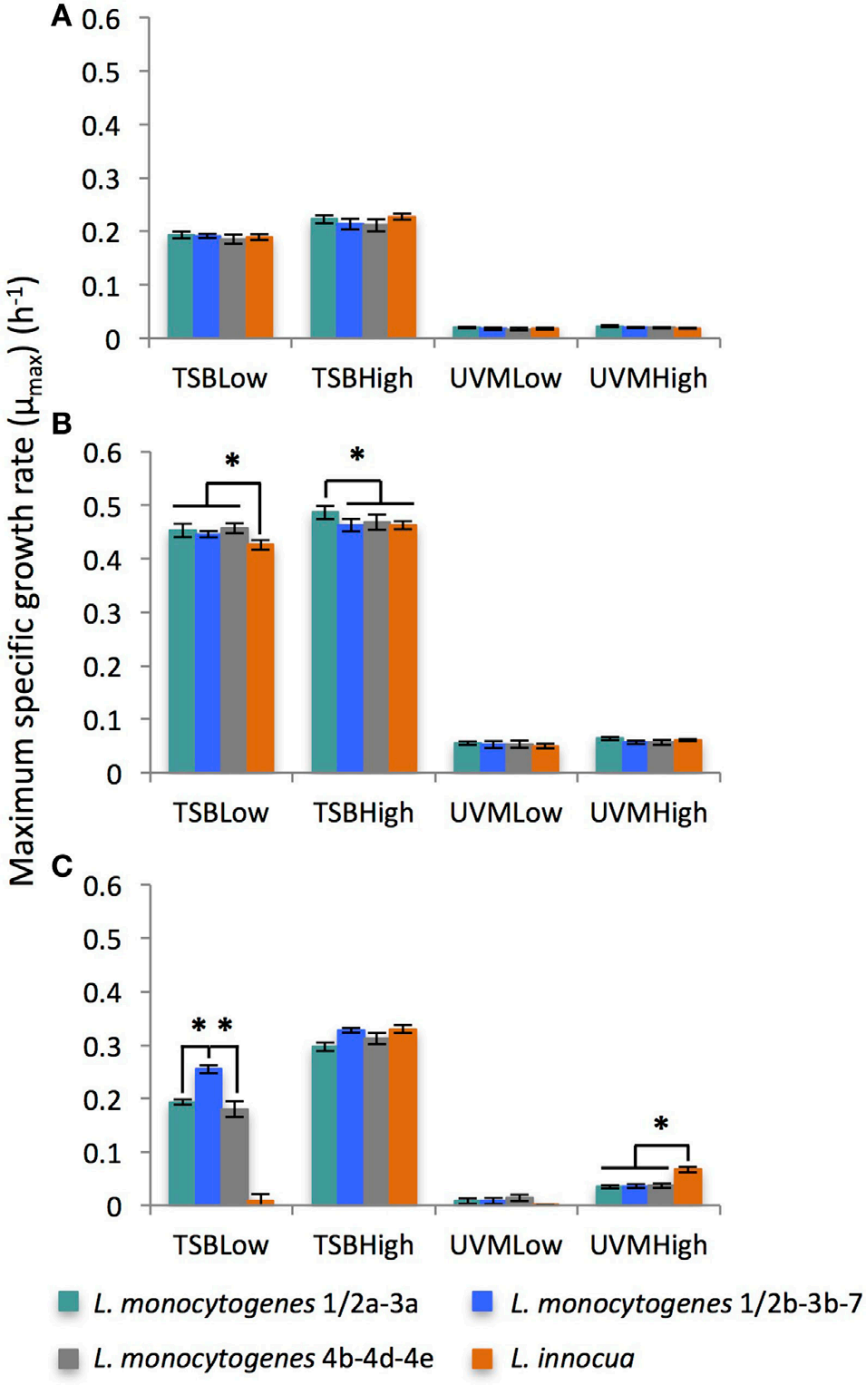
Average of maximum specific growth rate (μ_max_) of *Listeria monocytogenes* and *Listeria innocua* strains inoculated at low (10^2^ CFU/ml) and high (10^5^ CFU/ml) concentrations in TSB and UVM incubated at **(A)** 20°C, **(B)** 30°C, and **(C)** 42°C. *Significant differences between strains (*p* < 0.05). No growth observed for *L. innocua* in TSBLow and UVMLow at 42°C.

While many of the growth variables tested significantly effected μ_max_, by far the strongest association was to the enrichment medium (*F* = 2431, *p* < 0.0001), where the four *Listeria* strains grew faster in TSB than UVM (Figure 3). This result is in agreement with the general trend of *Listeria* strains from food origin showing a faster growth in general growth media (e.g., BHI, TSB + yeast extract) than *Listeria* enrichment media (UVM, Fraser, and Half-Fraser) (30, 33). We also observed that *Listeria* strains grew significantly faster at lower incubation temperatures, peaking at 30°C, and this effect was amplified in TSB medium (*F* = 81, *p* < 0.0001). This is in agreement with Duh and Schaffner (28), who showed that *Listeria* strains grew faster at temperatures below 41°C in general growth media (28). The growth variable with the weakest significant association to μ_max_ was initial inoculum concentration, where its effect were only observed in the 42°C treatments (*F* = 25, *p* > 0.0001).

#### Effect of Culture Medium, Inoculum Concentration, and Incubation Temperature on OD_max_

**Figure 4:**
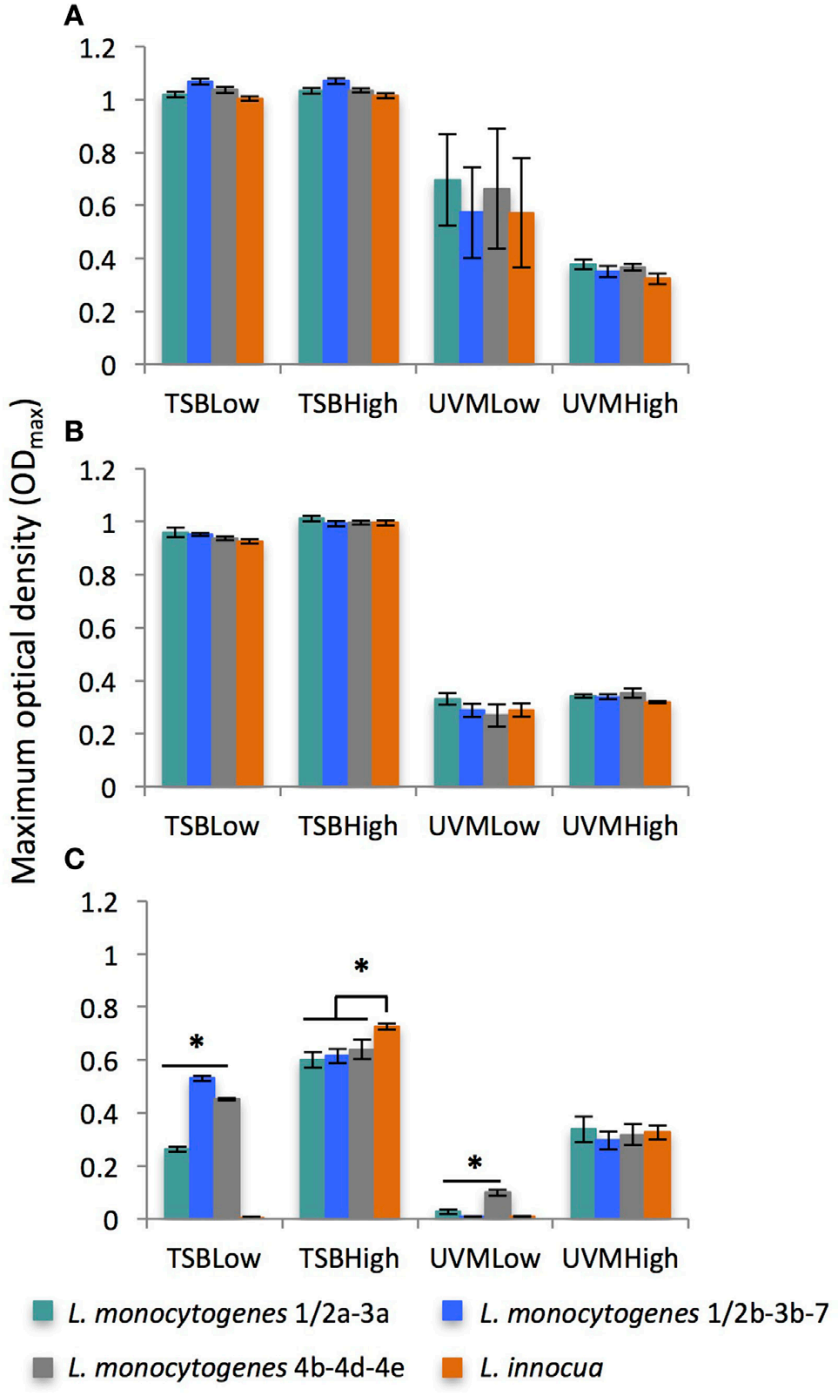
Average of maximum optical density (OD_max_) of *Listeria monocytogenes* and *Listeria innocua* strains inoculated at low (10^2^ CFU/ml) and high (10^5^ CFU/ml) concentrations in TSB and UVM incubated at **(A)** 20°C, **(B)** 30°C, and **(C)** 42°C. *Significant differences between strains (*p* < 0.05). No growth observed for *L. innocua* in TSBLow and UVMLow at 42°C.

As was observed for μ_max_, the enrichment medium was the dominant growth variable affecting OD_max_ (*F* = 1481, *p* < 0.0001) with significantly higher maximum optical densities found in the treatments grown in TSB (Figure 4). This is consistent with other data reporting a higher final cell density in the non-selective culture medium BHI than in selective enrichment media UVM or Half-Fraser (34, 54). For all *Listeria* strains, the OD_max_ significantly increased with decreasing incubation temperatures, especially in the UVM treatments (*F* = 193, *p* < 0.0001). Unlike λ or μ_max_, initial inoculum concentrations did not have a significant effect of OD_max_ overall (*F* = 0.01, *p* > 0.05), although limited effects were observed at treatments incubated at 42°C.

#### Comparing Monoculture Growth between *L. monocytogenes* and *L. innocua* Strains

While the general effects of the above variables on growth of *Listeria* spp. overall, the question of the differential effect between specific *Listeria* species still remained. While there were some exceptions, generally there were no significant differences between the three *L. monocytogenes* strains in terms of λ, μ_max_, or OD_max_, and regardless of enrichment media, the *L. innocua* strain was unable to grow at broiler body temperature (42°C) when the initial inoculum concentrations was 10^2^ CFU/ml (TSBLow and UVMLow). Significant differences in lag time between the three *L. monocytogenes* strains and the *L. innocua* strain varied based on the growth variables (Figure 2). At the average environmental and UVM enrichment temperatures (20 and 30°C, respectively), the lag time of *L. innocua* was significantly shorter than the *L. monocytogenes* strains in UVM, especially for low inoculum concentrations (*p* < 0.05; Figures 2A,B, respectively). However, at broiler body temperatures (42°C), *L. innocua* only grew in the high initial inoculum treatments (TSBHigh and UVMHigh), where there were no significant differences between the four *Listeria* strains (Figure 2C). No significant differences in λ between *L. innocua* and *L. monocytogenes* were found in any of the TSB treatments. Previous studies comparing the growth of *L. monocytogenes* and *L. innocua* strains mostly from food origins have reported shorter λ for *L. innocua* in Fraser (incubated at 30°C), and Half-Fraser (incubated at 37°C) enrichment media (31) and at lower incubation temperatures (≤8°C) (28).

While significant differences in μ_max_ were observed between the four *Listeria* strains used in this study, there were no consistent trends based on initial inoculum concentration, incubation temperature or enrichment medium (Figure 3). Only in two treatment combinations were there species-specific significant differences in μ_max_, with *L. monocytogenes* growing faster than *L. innocua* in TSBLow at 30°C (Figure 3B) and *L. innocua* growing faster than *L. monocytogenes* at 42°C in the UVMHigh treatment (Figure 3C). In contrast to previously reported findings, there were no significant differences found in μ_max_ between the *L. monocytogenes* and *L. innocua* isolates in UVM at 30°C, conditions used for the *Listeria* enrichment process (28, 31, 33). However, the studies comparing the generation time or the growth rate have shown a faster growth of *L. innocua* compared with *L. monocytogenes* at temperatures below 40°C only in certain culture media (28, 31, 33), which may explain the similar μ_max_ between *L. innocua* and *L. monocytogenes* in our study. In addition, a high level of heterogeneity in growth behavior within *L. innocua* and *L. monocytogenes* strains can lead to equivalent μ_max_ between the slowest *L. innocua* and the fastest *L. monocytogenes* (30, 55).

There were very few strain-specific differences in the maximum OD (OD_max_) for any of the growth variables, with the significant differences found at 42°C (Figure 4C). Among those difference, only under one treatment condition (TSBHigh) were there significant differences between *L. monocytogenes* and *L. innocua*, so overall the maximum cell density in culture was unaffected by the *Listeria* species. No differences were observed between the OD_max_ of *L. innocua* and *L. monocytogenes* species in UVM at 30°C as reported in studies using Half-Fraser and Fraser (30, 31). However, these results are highly dependent on the experiment and opposite trends are also reported showing an higher final population density of *L. innocua* than *L. monocytogenes* in enrichment media (29, 54).

### Differential Growth of *L. monocytogenes* 1/2a-3a and *L. innocua* in Coculture Growth Experiments

**Figure 5:**
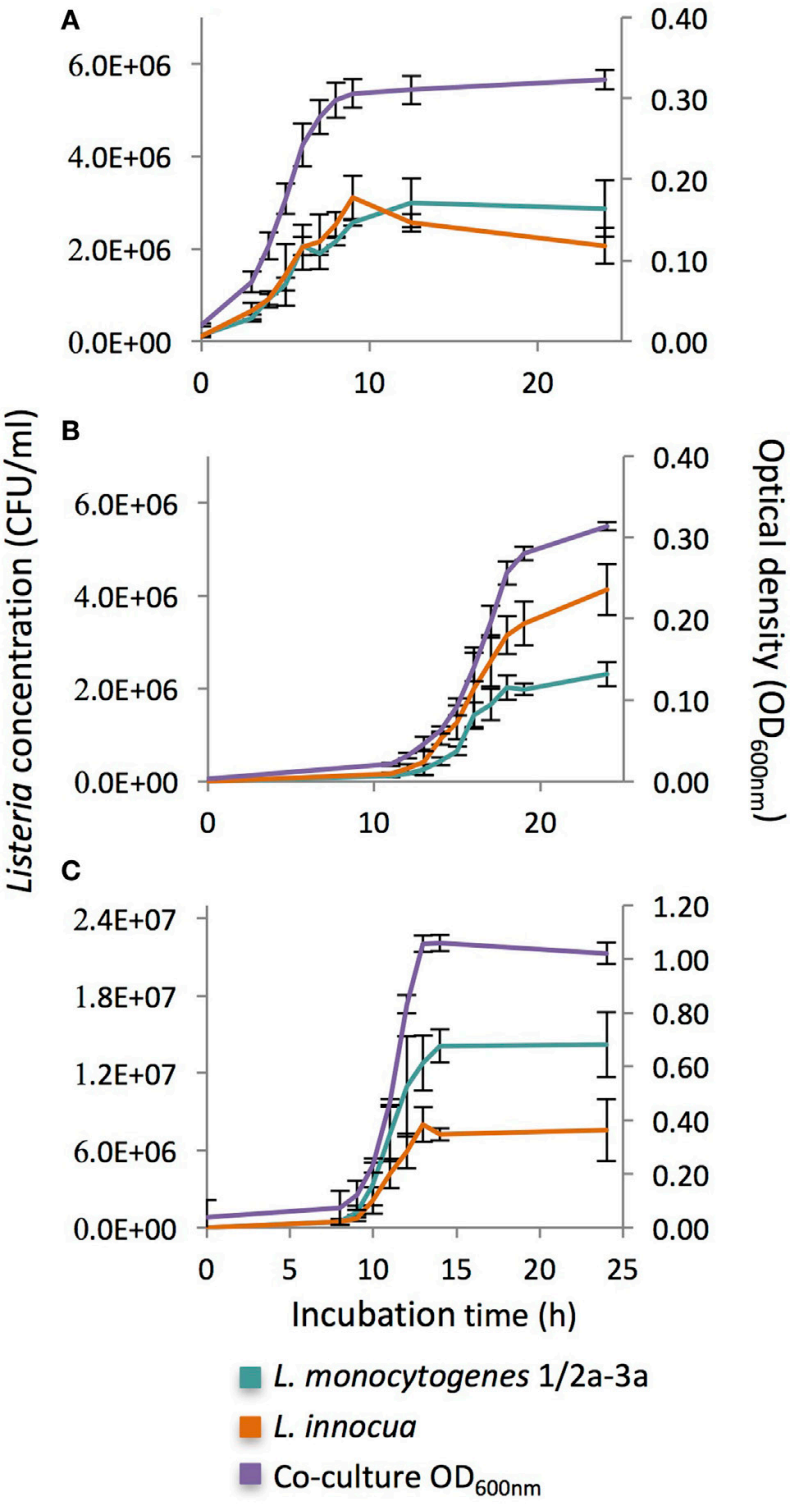
Growth curves of cocultures of *Listeria monocytogenes* serovar groups 1/2a-3a and *Listeria innocua* at 30°C inoculated at **(A)** high initial concentrations (10^5^ CFU/ml) in UVM, **(B)** low initial concentrations in UVM (10^2^ CFU/ml), and **(C)** low initial concentrations (10^2^ CFU/ml) in TSB.

Using the cultural conditions for the initial enrichment step for the USDA-FSIS MLG 8.10 *L. monocytogenes* enrichment method (UVM, 30°C) we found that *L. innocua* exhibited a significantly shorter lag time than the *L. monocytogenes* strains in monocultures, especially for low initial inoculum concentrations (10^2^ CFU/ml). To determine if *L. innocua* has any direct competitive growth advantages over *L. monocytogenes* in UVM, coculture experiments were performed using the *L. innocua* strain and the *L. monocytogenes* 1/2a-3a strain (the most prevalent serovar group from the farm surveys). When both strains were inoculated into the coculture at 10^5^ CFU/ml (Figure 5A), there were no significant differences in λ, μ_max_, or stationary phase cell density (similar to OD_max_), although *L. monocytogenes* densities did begin to exceed *L. innocua* cell densities after 24 h. When both strains started at the lower inoculum level (10^2^ CFU/ml), while λ and μ_max_ were similar, *L. innocua* reached a significantly higher stationary phase cell density compared with *L. monocytogenes* (*F* = 31, *p* < 0.01; Figure 5B). Conversely, when the cocultures inoculated at the lower initial concentrations were grown in TSB, *L. monocytogenes* demonstrated significantly higher stationary phase cell densities compared with *L. innocua* (*F* = 19, *p* < 0.05), with *L. monocytogenes* cell densities being ~3× greater than *L. innocua* (Figure 5C). When comparing the growth curve parameters among the three coculture experiments, only the stationary phase cell density was significantly effected at the species-level (Table 2).

**Table 2:** Growth parameters lag time (λ), maximum specific growth rate (μ_max_) and stationary phase cell density for triplicate cocultures studies of *Listeria monocytogenes* and *Listeria innocua* grown at 30°C and inoculated at two inoculum ratios (Low:Low and High:High) in two different growth media (UVM and TSB).

Growth medium	Inoculum ratio (*L. monocytogenes*: *L. innocua*)^a^	*Listeria* species	λ (h)^b^	μ_max_ (h^**−**1^)^b^	Stationary phase cell density (log_10_ CFU/ml)^b^
UVM	High:High	*monocytogenes*	1.95 ± 0.17^A^	5.65 ± 0.04^A^	6.47 ± 0.02^A^
		*Innocua*	2.56 ± 0.43^A^	5.82 ± 0.06^A^	6.41 ± 0.02^A^
UVM	Low:Low	*monocytogenes*	12.74 ± 0.45^B^	5.68 ± 0.11^A^	6.38 ± 0.05^AB^
		*innocua*	13.20 ± 0.45^B^	5.79 ± 0.07^A^	6.64 ± 0.07^B^
TSB	Low:Low	*monocytogenes*	9.27 ± 0.45^C^	6.64 ± 0.11^B^	7.17 ± 0.06^C^
		*innocua*	9.24 ± 0.76^C^	6.44 ± 0.12^B^	6.89 ± 0.09^B^

*^a^High = 10^5^ CFU/ml; Low = 10^2^ CFU/ml*.

*^b^Superscript letters “A–C” indicate significant differences (*p* < 0.05) in a single column*.

Unlike the monoculture results using the UVM protocol from the USDA-FSIS MLG 8.10 method, no significant lag time difference was observed between *L. innocua* and *L. monocytogenes* at low initial inoculum concentrations; however, *L. innocua* still maintained a competitive growth advantage under these cultural conditions represented by significantly higher stationary phase cell densities (Table 2; Figure 5B). When grown under the same conditions in TSB (Figure 5C), *L. monocytogenes* grew at significantly higher levels than *L. innocua*, indicating that there is a enrichment media-specific effect on *Listeria* growth within these cocultures. When looking at the differences in stationary phase cell densities across all cocultures, there was no significant difference for *L. innocua* between UVM and TSB in the Low:Low cocultures, but there was over a 1 log reduction in stationary phase cell density for *L. monocytogenes* grown in TSB (7.17 ± 0.06 log_10_ CFU/ml) compared with UVM (6.38 ± 0.05 log_10_ CFU/ml) under those growth conditions (Table 2). While it appears that *L. innocua* has a competitive growth advantage in UVM with low initial inoculum concentrations, it is possible that this advantage comes more from a disadvantage of *L. monocytogenes* growing under these conditions, rather than a specific advantage that *L. innocua* possesses, and previous studies have shown that enrichment/culture media can differentially effect *L. innocua* and *L. monocytogenes* (28, 31, 33).

However, considering the UVM enrichment is the first of two enrichments in the USDA-FSIS MLG 8.10 protocol, having significantly higher densities of *L. innocua* compared with *L. monocytogenes* would artificially increase the likelihood of isolating *L. innocua* from samples with equivalent levels of *L. innocua* and *L. monocytogenes*. Considering the UVM enrichment is used within the USDA-FSIS MLG 8.10 method, and USDA-FSIS is responsible for the testing of foodborne pathogens from broiler production farms and processing plants, this enrichment bias could potentially or partially explain the prevalence of *L. innocua* as the dominant *Listeria* spp. found on poultry farms (15, 23–26).

## Conclusion

In our study, we found that *L. innocua* is more prevalent than the foodborne pathogen *L. monocytogenes* in soil and feces samples collected from pastured poultry farms, which is consistent with conventional poultry farms. Mono- and coculture growth experiments showed that under cultural conditions used in the first enrichment step of the USDA-FSIS MLG 8.10 *L. monocytogenes* method (UVM, 30°C), *L. innocua* had a significantly shorter lag phase (monoculture) and a significantly higher stationary phase cell density (coculture) compared with *L. monocytogenes*; these growth advantages occurred at low initial inoculum concentrations simulating the low levels of *Listeria* species encountered in the environment. Based on these results, it is possible that UVM enrichment medium either preferentially supports *L. innocua* growth over *L. monocytogenes*, or preferentially restricts *L. monocytogenes* growth, and that this enrichment step may be biasing the recovery of *L. innocua* over *L. monocytogenes* from live production samples. Considering the public health importance of accurately identifying the source of *L. monocytogenes* outbreaks, future work will need to understand the cultural and molecular mechanisms of this preferential *L. innocua* growth in UVM, and alternative enrichment methods for *L. monocytogenes* may need to be considered.

## Author Contributions

AL and MR helped to develop experiments, analyze data, and prepare manuscript. ML helped in data analysis and manuscript preparation.

## Conflict of Interest Statement

The authors declare that the research was conducted in the absence of any commercial or financial relationships that could be construed as a potential conflict of interest. The reviewer KG declared a shared affiliation, with no collaboration, with the authors to the handling editor.
