# Supplementary material for: The Distribution of Listeria in Pasture-Raised Broiler Farm Soils Is Potentially Related to University of Vermont Medium Enrichment Bias toward Listeria innocua over Listeria monocytogenes
Source: Front Vet Sci. 2017 Dec 21;4:227. doi: 10.3389/fvets.2017.00227 (PMC5742580; doi:10.3389/fvets.2017.00227)
Supplement: Supplementary file 1 [file Table_1.docx]

| **Sources of variation** | **df** | **Sum of squares** | **F value** |
| --- | --- | --- | --- |
| Temperature | 2 | 10744 | 173.57 *** |
| Strain | 3 | 523 | 5.63 *** |
| Media | 1 | 1122 | 36.26 *** |
| Concentration | 1 | 24192 | 801.03 *** |
| Temperature*Strain | 6 | 415 | 2.23 * |
| Temperature*Media | 2 | 1383 | 22.34 *** |
| Strain*Media | 3 | 106 | 1.14 |
| Temperature*Concentration | 2 | 4753 | 76.78 *** |
| Strain*Concentration | 3 | 261 | 2.81 * |
| Media*Concentration | 1 | 1856 | 59.96 *** |
| Temperature*Strain*Media | 6 | 32 | 0.17 |
| Temperature*Strain*Concentration | 6 | 525 | 2.83 * |
| Temperature*Media*Concentration | 2 | 1402 | 22.64 *** |
| Strain*Media*Concentration | 3 | 44 | 0.47 |
| Temperature*Strain*Media*Concentration | 6 | 69 | 0.37 |

Table S1: Analysis of variance (4-way ANOVA) for lag time (λ) values determined from growth curves of pure cultures of *L. monocytogenes* serotype 1/2a-3a, *L. monocytogenes* serotype 1/2b-3b, *L. monocytogenes* serotype 4b-4c-4d and *L. innocua* strains inoculated at low (10^2^ cells/ml) and high (10^5^ cells/ml) concentration in TSB and UVM media and incubated at 20°C, 30°C and 42°C.

df: degrees of freedom, F value: Fisher’s F.

*** p<0.001

** p<0.01

* p<0.05

Table S2: Analysis of variance (4-way ANOVA) for maximum specific growth rate (μmax) values determined from growth curves of pure cultures of *L. monocytogenes* serotype 1/2a-3a, *L. monocytogenes* serotype 1/2b-3b, *L. monocytogenes* serotype 4b-4c-4d and *L. innocua* strains inoculated at low (10^2^ cells/ml) and high (10^5^ cells/ml) concentration in TSB and UVM media and incubated at 20°C, 30°C and 42°C.

| **Sources of variation** | **df** | **Sum of squares** | **F value** |
| --- | --- | --- | --- |
| Temperature | 2 | 1.079 | 144.64 *** |
| Strain | 3 | 0.021 | 1.84 |
| Media | 1 | 9.101 | 2440 *** |
| Concentration | 1 | 0.214 | 57.42 *** |
| Temperature*Strain | 6 | 0.008 | 2.10 |
| Temperature*Media | 2 | 0.303 | 81.26 *** |
| Strain*Media | 3 | 0.010 | 2.77 * |
| Temperature*Concentration | 2 | 0.093 | 24.96 *** |
| Strain*Concentration | 3 | 0.016 | 4.26 ** |
| Media*Concentration | 1 | 0.092 | 24.57 *** |
| Temperature*Strain*Media | 6 | 0.065 | 2.91 ** |
| Temperature*Strain*Concentration | 6 | 0.084 | 3.77 ** |
| Temperature*Media*Concentration | 2 | 0.070 | 9.42 *** |
| Strain*Media*Concentration | 3 | 0.027 | 2.41 |
| Temperature*Strain*Media*Concentration | 6 | 0.040 | 1.78 |

df: degrees of freedom, F value: Fisher’s F.

*** p<0.001

** p<0.01

* p<0.05

Table S3: Analysis of variance (4-way ANOVA) for maximum optical density values (ODmax) determined from growth curves of pure cultures of *L. monocytogenes* serotype 1/2a-3a, *L. monocytogenes* serotype 1/2b-3b, *L. monocytogenes* serotype 4b-4c-4d and *L. innocua* strains inoculated at low (10^2^ cells/ml) and high (10^5^ cells/ml) concentration in TSB and UVM media and incubated at 20°C, 30°C and 42°C.

| **Sources of variation** | **df** | **Sum of squares** | **F value** |
| --- | --- | --- | --- |
| Temperature | 2 | 9.48 | 192.61 *** |
| Strain | 3 | 0.45 | 6.15 *** |
| Media | 1 | 36.45 | 1480.95 *** |
| Concentration | 1 | 0.00 | 0.01 |
| Temperature*Strain | 6 | 0.50 | 3.4 ** |
| Temperature*Media | 2 | 2.25 | 45.64 *** |
| Strain*Media | 3 | 0.06 | 0.79 |
| Temperature*Concentration | 2 | 1.60 | 32.5 *** |
| Strain*Concentration | 3 | 0.38 | 5.2 ** |
| Media*Concentration | 1 | 0.97 | 39.6 *** |
| Temperature*Strain*Media | 6 | 0.04 | 0.26 |
| Temperature*Strain*Concentration | 6 | 0.96 | 1.3x10-6 *** |
| Temperature*Media*Concentration | 2 | 0.18 | 0.03 * |
| Strain*Media*Concentration | 3 | 0.02 | 0.80 |
| Temperature*Strain*Media*Concentration | 6 | 0.10 | 0.66 |

df: degrees of freedom, F value: Fisher’s F.

*** p<0.001

** p<0.01

* p<0.05
